# Supplementary figures and images for: No effect of a dairy-based, high flavonoid pre-workout beverage on exercise-induced intestinal injury, permeability, and inflammation in recreational cyclists: A randomized controlled crossover trial
Source: PLoS One. 2022 Nov 29;17(11):e0277453. doi: 10.1371/journal.pone.0277453 (PMC9707743; doi:10.1371/journal.pone.0277453)

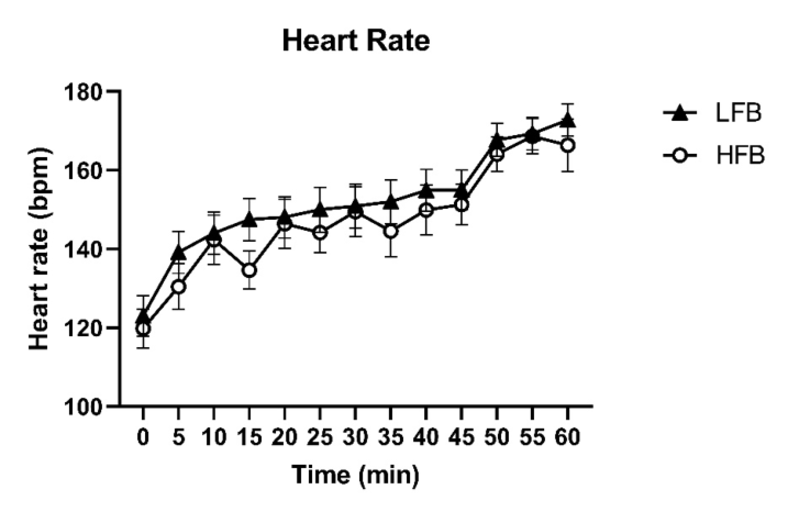

Supplement: S1 Fig — (TIF) [file pone.0277453.s002.tif]
