# Supplementary material for: No effect of a dairy-based, high flavonoid pre-workout beverage on exercise-induced intestinal injury, permeability, and inflammation in recreational cyclists: A randomized controlled crossover trial
Source: PLoS One. 2022 Nov 29;17(11):e0277453. doi: 10.1371/journal.pone.0277453 (PMC9707743; doi:10.1371/journal.pone.0277453)
Supplement: S1 Table — (DOCX) [file pone.0277453.s003.docx]

| **Ingredient** | **HFB % (w/w)** | **Ingredient** | **LFB % (w/w)** |
| --- | --- | --- | --- |
| 2% Milk | 78.1 | 2% Milk | 78.2 |
| Sugar | 8.6 | Sugar | 8.6 |
| Maltodextrin | 8.6 | Maltodextrin | 8.6 |
| Blueberry powder | 2.4 | Placebo powder | 2.4 |
| Cocoa powder | 1.6 | Alkalized cocoa powder | 1.6 |
| Whey protein isolate | 0.6 | Whey protein isolate | 0.6 |
| Green tea powder | 0.1 | Green tea powder | 0 |
