# Supplementary material for: No effect of a dairy-based, high flavonoid pre-workout beverage on exercise-induced intestinal injury, permeability, and inflammation in recreational cyclists: A randomized controlled crossover trial
Source: PLoS One. 2022 Nov 29;17(11):e0277453. doi: 10.1371/journal.pone.0277453 (PMC9707743; doi:10.1371/journal.pone.0277453)
Supplement: S2 Table — (DOCX) [file pone.0277453.s004.docx]

|  | **5 min** | **15 min** | **25 min** | **35 min** |
| --- | --- | --- | --- | --- |
| HFB | 2.9 ± 0.3^a^ | 3.2 ± 0.3^ab^ | 3.8 ± 0.4^b^ | 4.0 ± 0.4^b^ |
| LFB | 2.7 ± 0.4^a^ | 3.3 ± 0.4^ab^ | 3.6 ± 0.5^b^ | 3.9 ± 0.5^b^ |
| HFB-LFB | 0.3 ± 0.2 | -0.1 ± 0.2 | 0.3 ± 0.2 | 0.1 ± 0.3 |

RPE scale 0-10. Values with different superscripts differed significantly (p < 0.05).
