# Supplementary material for: No effect of a dairy-based, high flavonoid pre-workout beverage on exercise-induced intestinal injury, permeability, and inflammation in recreational cyclists: A randomized controlled crossover trial
Source: PLoS One. 2022 Nov 29;17(11):e0277453. doi: 10.1371/journal.pone.0277453 (PMC9707743; doi:10.1371/journal.pone.0277453)
Supplement: S3 Table — (DOCX) [file pone.0277453.s005.docx]

|  | **10 min** | **20 min** | **30 min** | **40 min** |
| --- | --- | --- | --- | --- |
| HFB | 29.9 ± 1.4^a^ | 30.2 ± 1.3^b^ | 30.6 ± 1.4^b^ | 30.5 ± 1.3^b^ |
| LFB | 29.6 ± 1.4^a^ | 30.2 ± 1.5^b^ | 30.4 ± 1.4^b^ | 30.4 ± 1.4^b^ |
| HFB-LFB | 0.37 ± 0.58 | -0.01 ± 0.46 | 0.23 ± 0.33 | 0.10 ± 0.25 |

Values with different superscripts differed significantly (p < 0.05).
