# Supplementary material for: No effect of a dairy-based, high flavonoid pre-workout beverage on exercise-induced intestinal injury, permeability, and inflammation in recreational cyclists: A randomized controlled crossover trial
Source: PLoS One. 2022 Nov 29;17(11):e0277453. doi: 10.1371/journal.pone.0277453 (PMC9707743; doi:10.1371/journal.pone.0277453)
Supplement: Attachment — Submitted filename: Response_to_Reviewers.docx [file pone.0277453.s007.docx]

**Reviewer/Editor Comment:**

In the previous revision round, reviewer 2 mentioned that the data provided in Figure 3 appear to represent the total distance covered over the full 1-hour cycling protocol (so the 45 min at 70%-power, plus the 15 min all-out TT). I understand that point, since the participants were not particularly well-trained average VO2max of 43 ml/kg/min, max power output approximately 225 @). With such a training status it seems impossible to cycle 19 km in 15 minutes!! Also, the power output during the 'all-out' 15 min-TT does not seem to be much different from the 70% intensity during the first 45 minutes, and would not allow to be translated to 19 km. The reviewer therefore assumed that the data in figure 3A and 3B are actually the data from the full 60-minutes of cycling rather than only the 15-min TT. Although 19 km covered distance within an hour cycling would be on the low side, the same distance within only 15 min seems impossible. This inconsistency should be clarified.

The authors have now responded by stating that these data are actually from the 15-min TT, but then the 19 km covered distance does not make much sense, as they would be cycling at 76 km/h!! Please double-check the calculations for covered distance and power output and report those data only for the 15-min TT in figure 3.

**Response:**

Somehow, we missed this. The reviewer is correct. The distance was for the whole time on the bike, and not only the time trial. We have corrected this in the resubmitted manuscript. The work reported, however, was correct. These edits do not change the outcome or study findings.

We appreciate the comments and are a bit embarrassed we missed this the last time. d
